# Rate of avascular necrosis after fracture dislocations of the proximal humerus: Timing of surgery

**DOI:** 10.1007/s11678-018-0452-6

**Published:** 2018-03-07

**Authors:** Marc Schnetzke, Julia Bockmeyer, Markus Loew, Stefan Studier-Fischer, Paul-Alfred Grützner, Thorsten Guehring

**Affiliations:** 1 BG Trauma Center Ludwigshafen, Clinic for Trauma and Orthopaedic Surgery at the University of Heidelberg, Ludwig-Guttmann-Straße 13, 67071 Ludwigshafen on the Rhine, Germany; 2 ATOS Clinic Heidelberg, German Joint Center Heidelberg, Heidelberg, Germany

**Keywords:** Osteonecrosis, Shoulder fractures, Shoulder joint, Treatment outcome, Revision surgery, Osteonekrose, Schulterfrakturen, Schultergelenk, Therapieergebnis, Revisionsoperation

## Abstract

**Background:**

Avascular necrosis (AVN) of the humeral head is a severe complication after proximal humerus fracture dislocations, and leads to a poorer clinical outcome and subsequent revision surgeries. The aim of the current study was to analyze the influence of time to surgery on the AVN rate after locked plating of dislocation fractures of the proximal humerus.

**Patients and methods:**

This retrospective study included 30 patients with a mean age of 63 ± 14 years with dislocation fractures of the proximal humerus type B3 or C3 according the AO/OTA classification. The rates of AVN of the humeral head were determined clinically and radiographically. In addition, the clinical outcome was determined using the Constant score (CS), the age- and sex-adjusted Constant score (CS%), Disabilities of the Arm, Shoulder, and Hand (DASH) score, the range of motion, and complication and revision rates. Patients were subdivided into groups of subjects operated on early (≤48 h after trauma) and those with late surgery (>48 h after trauma), and the relative risk (RR) for complications and revisions was determined for both groups.

**Results:**

After a mean follow-up of 37 months (range: 12–66 month) the mean CS% was 60 ± 24 and the mean DASH score was 32 ± 24 points. Ten patients (33%) developed a symptomatic AVN, and ten patients underwent revision surgery. Early surgery was performed on 25 patients while five patients underwent late surgery. After late surgery, all five patients developed AVN, and patients had a fivefold increased RR for AVN (*p* = 0.002) and subsequent associated surgical revision (RR = 3.3, *p* = 0.031).

**Conclusion:**

In fracture dislocations of the proximal humerus, early surgery within 48 h of trauma significantly decreases the risk of AVN and subsequent surgery.

## Zusammenfassung

**Hintergrund:**

Die Humeruskopfnekrose (HKN) stellt eine schwerwiegende Komplikation bei der Behandlung von Humeruskopffrakturen dar. Die HKN geht meist mit einem unbefriedigenden klinischen Ergebnis und der Notwendigkeit von Revisionsoperationen einher. Das Ziel der aktuellen Studie war es, den Einfluss des Operationszeitpunkts auf die Rate der HKN nach winkelstabiler Plattenosteosynthese von Humeruskopfluxationsfrakturen zu untersuchen.

**Material und Methoden:**

In diese retrospektive Studie wurden 30 Patienten (Durchschnittsalter 63 ± 14 Jahre) mit einer Humeruskopfluxationsfraktur Typ 11B3 oder 11C3 nach der OTA/AO-Klassifikation (Orthopedic Trauma Association/Arbeitsgemeinschaft für Osteosynthesefragen) eingeschlossen. Das Vorliegen einer HKN wurde anhand klinischer und radiologischer Kriterien bestimmt. An klinischen Parametern wurden der Constant Score (CS), der geschlechts- und altersadaptierte Constant Score (CS%), Disabilities of the Arm, Shoulder and Hand (DASH) Score und das Bewegungsausmaß erfasst. Zusätzlich wurden Komplikationen und Revisionsoperationen dokumentiert. Anhand des Operationszeitpunkts nach dem Unfallereignis wurden die Patienten in 2 Gruppen unterteilt (frühe Operation: ≤48 h vs. späte Operation: >48 h nach Trauma). Das relative Risiko (RR) für Komplikationen und Revisionen in beiden Gruppen wurde ermittelt.

**Ergebnisse:**

Nach einem mittleren Nachbeobachtungszeitraum von 37 Monaten (12–66 Monate) betrug der CS% 60 ± 24 und der DASH Score 32 ± 24 Punkte. Eine HKN entwickelten 10 Patienten, und bei 10 Patienten wurde eine Revisionsoperation durchgeführt. Frühzeitig (≤48 h) nach Trauma wurden 25 von 30 Patienten und 5 Patienten >48 h nach Trauma operiert. Alle 5 Patienten mit spätem Operationszeitpunkt entwickelten eine HKN. Patienten mit einem späten Operationszeitpunkt hatten ein erhöhtes RR für die Entstehung einer HKN (RR 5,0; *p* = 0,002) und ein erhöhtes RR für die Notwendigkeit einer Revisionsoperation (RR = 3,3, *p* = 0,031).

**Schlussfolgerung:**

Bei der Osteosynthese von Humeruskopfluxationsfrakturen führt ein früher Operationszeitpunkt innerhalb von 48 h nach Trauma zu einer signifikanten Reduktion der HKN-Rate.

The treatment of dislocation fractures of the proximal humerus is challenging. Complications such as avascular necrosis of the humeral head can lead to worse outcomes. The relationship between timing of surgery and the development of the avascular necrosis in dislocation fractures of the proximal humerus has not been investigated thoroughly to date

## Introduction

The fracture dislocation of the proximal humerus represents a rare injury, and in these patients operative treatment is required owing to dislocation of the humeral head [9, 20]. High rates of complications after reconstruction of proximal humerus fractures regardless of the method of fixation are reported [18, 19]. Avascular necrosis (AVN) of the humeral head is one of the most feared short-term complications after reconstructive surgery of proximal humerus fractures [6, 7]. AVN leads to worse clinical outcome and subsequent revision surgery in most patients [13]. In fracture dislocations an even higher risk of AVN might be considered owing to the limited blood supply of the humeral head [7]. Therefore, the time spent waiting for surgery might be one important aspect in fracture dislocations of the humeral head with respect to AVN rate. Several authors have postulated that stable internal fixation promotes revascularization of the humeral head after fracture [7, 21]. Early stabilization of these fractures could therefore be expected to reduce ischemic time for the humeral head and perhaps the rate of AVN. Thus, the aim of the current study was to investigate the correlation between time to surgery and AVN rate in fracture dislocations of the proximal humerus. We hypothesized that patients with early reconstructive surgery within 48 h of trauma could have significantly lower rates of AVN of the humeral head compared with patients who underwent late surgery (>48 h).

## Methods

This retrospective study was enrolled at a level I trauma center after approval of the local ethics committee (No. 837.503.14/9742). Inclusion criteria were age ≥18 years, dislocation fracture of the humeral head type B3 or C3 according to the AO/Orthopaedic Trauma Association (OTA) classification system [10], treatment with locking plate fixation (PHILOS, Synthes, Davos, Switzerland), a minimum follow-up of 1 year, and written informed consent. According to the classification system, patients were only included in the case of complete dislocation of the humeral head from the glenoid. Between January 2008 and October 2014, 39 patients were identified. Nine patients could not be reached or were not able to come to the follow-up examination. Finally, 30 patients (77%) were included in this study. The mean age of the study population was 63 ± 14 years (range: 34–86 years); 12 patients (40%) were male and 18 patients (60%) were female. The right side was injured in 14 patients (47%) and the left side in 16 patients (53%). According to the AO/OTA classification system, 28 patients (93%) had an 11C3 fracture type and two patients (7%) had an 11B3 fracture type. Of the patients, 13 (43%) had at least one comorbidity, with the most frequent being hypertension (*n* = 11; 37%) and diabetes (*n* = 4; 13%).

### Treatment protocol and timing of surgery

In all patients, a surgical approach was required to reduce the dislocated humeral head and to repair the fracture. The surgical treatment was planned as soon as possible for all patients. However, five patients underwent surgery with open reduction more than 48 h after trauma, as they were referred late from another hospital. In these five patients, closed reduction was not performed or not possible before surgical treatment. We therefore divided the study population into patients with early surgery (within 48 h of trauma) and those with late surgery (>48 h after trauma).

In all patients, operative treatment was performed with a locking plate fixation (PHILOS, Synthes, Davos, Switzerland) through a deltoideal pectoral approach. The plate was positioned 3–5 mm laterally of the long head of the biceps. The chosen head screws were as long as possible and were subjected to careful intraoperative fluoroscopic evaluation. Every effort was made to place the calcar screws for additional medial support. Patients were immobilized in a sling for 1–2 weeks postoperatively. Functional exercises were started after a correct position of the humeral head in the joint was determined on radiographs obtained on the second postoperative day. Full weight-bearing was allowed after 6 weeks.

### Follow-up protocol

The clinical examination was performed by one independent examiner. In all patients the range of motion, Constant score (CS), age- and sex-adjusted Constant score (CS%), and the Disabilities of the Arm, Shoulder, and Hand (DASH) score were investigated at final follow-up. The surgical revision rates and reasons were also recorded. The influence of the timing of surgery was analyzed: Surgery within 48 h of trauma was defined as *early* and surgery after >48 h was defined as *late* according to previous studies [11, 16]. The immediate postoperative radiographs were analyzed with regard to the quality of fracture and humeral head reduction according to a previous study from our study group [15]. Briefly, the reduction of the head–shaft angle (normal, varus, valgus), head–shaft dislocation (no, <5 mm, >5 mm), and displacement of the major tubercle (no, <5 mm, >5 mm) were analyzed. Overall, the reposition was defined as *anatomical* quality of reduction in cases of head-shaft angles of 110–150°, <5 mm head-shaft dislocation, and <5 mm displacement of the major tubercle on the postoperative radiographs. In the case of varus <110° or valgus >150°, >5 mm head–shaft dislocation, or >5 mm displacement of the major tubercle, the reposition was defined as *poor *quality of reduction. At final follow-up, radiographs were obtained if the patient reported pain, or if a decrease in range of motion or a low functional result was identified with respect to ethics committee objections. The follow-up radiographs were analyzed for complications related to the fracture fixation such as AVN, secondary fracture dislocation, screw cut out, or non-union. The AVN was classified according to the system of Cruess [5].

### Statistical analysis

Mean and standard deviation (SD) were calculated for continuous variables. Differences between the two groups of patients were calculated using the Mann-Whitney *U* test. To identify prognostic factors for the development of AVN or the need for revision surgeries, relative risk (RR) and the results of Fisher’s exact test were determined for the time of surgery (late surgery >48 h). In addition, the influence of four independent factors (female gender, age >65 years, >1 comorbidity, poor fracture reduction) on the occurrence of AVN and the need for revision surgery was determined. A two-tailed *p* value of <0.05 was considered to show a significant difference. SPSS software (version 23.0; IBM) was used for the analysis.

## Results

At final-follow up after 37 months (range: 12–66 months), the mean CS was 52 ± 22 (range: 20–77), the CS% was 60 ± 24 (range: 27–100), and the DASH score was 32 ± 24 (range: 2–73). Mean abduction was 83° ± 42 (range: 15–165°), flexion was 95° ± 37 (range: 45–165°), and external rotation was 17° ± 17 (range: 0–40°). Ten patients (33%) developed an AVN of the humeral head and ten patients (33%) underwent revision surgery (Table 1).

**Table 1:** Complications and revisions in patients with early and late surgery

	Early surgery (*n* = 25)	Late surgery (*n* = 5)
*Complication, n (%)*		
Avascular necrosis	5 (20)	5 (100)
Infection	0	1 (20)
Non-union	2 (8)	0
Secondary fracture dislocation	5 (20)	1 (20)
*Revision surgery, n (%)*		
Revision to TSA/RSA	2 (8)	4 (80)
Implant removal with open surgical release	3 (12)	1 (20)

*TSA* total shoulder arthroplasty, *RSA* reverse shoulder arthroplasty

**Fig. 1:**
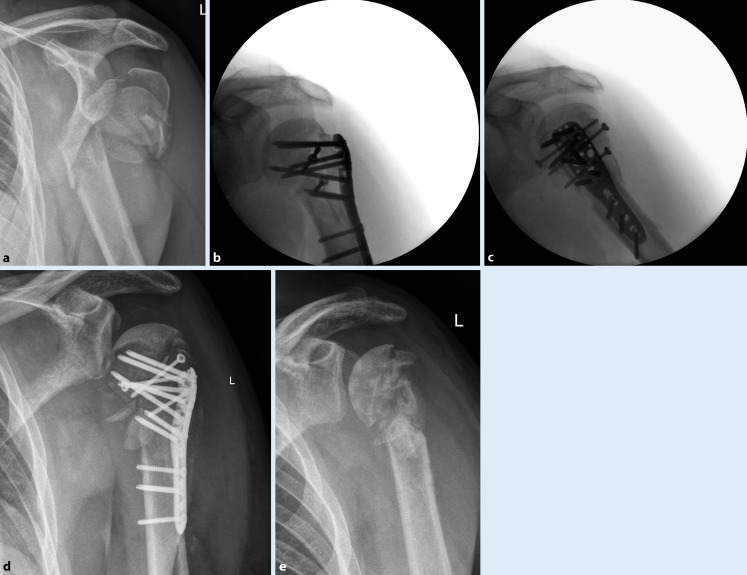
A 50-year-old patient with a dislocation fracture type 11C3 on the left side (**a**). Surgery was performed 3 days after trauma. Intraoperatively, the humeral head was completely dislocated from the glenoid. The intraoperative radiographs confirmed an anatomical fracture reduction (**b,** **c**). After 3 months, the patient complained of pain on movement and the radiograph showed a stage III avascular necrosis of the inferior part of the humeral head (**d**). The patient underwent revision surgery with implant removal alone (**e**)

Patients with AVN of the humeral head presented clinically with increased pain, and the diagnosis was made on the basis of such clinical signs and a change on the radiographs, which showed AVN stage II in three patients, stage III in five patients (Fig. 1), and stage IV in two patients. Detailed results of patients with AVN are shown in Table 2.

**Table 2:** Detailed results for patients with and without AVN

	With AVN (*n* = 10)	Without AVN (*n* = 20)
*Age (mean; range)*	64 (43–83)	62 (34–86)
*Time to surgery (n)*		
<48 h	5	20
>48 h	5	0
*Time to surgery (mean* *±* *SD)*	3.0 ± 3.0	0.4 ± 0.6
*Quality of reduction (n)*		
Anatomical	1	9
Poor	9	11
*Range of motion (°, range)*		
Abduction	65 (45–105)	88 (45–165)
Flexion	75 (25–115)	101 (45–165)
External rotation	13 (0–40)	18 (0–40)
*CS% (mean* *±* *SD)*	49 ± 7	64 ± 26
*Complications (n)*	10	5
*Revision surgery (n)*	7	3

*AVN* avascular necrosis, *CS%* age- and sex-adjusted Constant score, *SD* standard deviation

In all, 25 patients (83%) underwent early surgery within 48 h of trauma (range: 0–2 days) and five patients (17%) had late surgery, >48 h after trauma (range: 3–7 days). Patients with late surgery had more complications (100% vs. 48%; *p* = 0.042) and underwent more revision surgeries (100% vs. 20%; *p* = 0.018). In the late surgery group, five of five patients (100%) developed an AVN of the humeral head, and in the early group, five of 25 patients (20%) developed an AVN of the humeral head (*p* = 0.002; Table 3). Overall, surgery was performed after an average period of 1.3 ± 2.1 days after trauma. Patients with subsequent AVN underwent surgery after an average of 3.0 ± 3.0 days (range: 0–7 days) and patients without subsequent AVN after 0.4 ± 0.6 days (range: 0–2 days; *p* = 0.007).

**Table 3:** Analysis of risk factors for complications, avascular necrosis and revision surgeries

Risk factor	Avascular necrosis		Revision surgery	
	RR	*p*	RR	*p*
Female gender; *n* = 17	1.0	1.0	1.0	1.0
Age >65; *n* = 17	1.3	0.705	0.9	1.0
>1 comorbidity; *n* = 12	0.6	0.694	1.0	1.0
Late surgery (>48 h); *n* = 5	5.0	0.002	3.3	0.031
Poor reduction; *n* = 20	4.5	0.101	11.0	0.011

*RR* relative risk

According to our proposed criteria for quality of fracture reduction, anatomical reconstruction was achieved in ten patients (33%), whereas 20 patients (67%) had a poor quality of reduction. Nine out of 20 patients (45%) with poor quality of fracture reduction and one out of ten patients (10%) with anatomical fracture reduction developed an AVN of the humeral head. Overall, two out of ten patients with anatomical reduction developed complications (20%) and 13 out of 20 patients with poor quality of fracture reduction (65%) developed complications (*p* = 0.050). None of the patients with anatomical fracture reduction and ten out of 20 patients (50%) with poor fracture reduction underwent revision surgery (*p* = 0.011). Poor fracture reduction was associated with a 4.5-fold RR for development of AVN (10% vs. 45%; *p* = 0.101). Other factors such as female gender, age >65, and more than one comorbidity did not increase the risk for complications or revision surgeries.

## Discussion

The most important finding of the current study is that late surgery (>48 h) after injury as well as poor quality of fracture reduction significantly increase the risk of AVN and the need for revision surgery after reconstruction of fracture dislocations of the humeral head. Five patients were treated late (>48 h) after injury and all of these patients developed an AVN of the humeral head, leading to revision surgery with secondary total shoulder arthroplasty in four patients and implant removal in one patient (Fig. 1). In the group of patients with early surgery within 48 h of injury, only five of 25 patients (20%) developed an AVN.

Limited reports with only a few patients included are available that determined the influence of time to surgery on the occurrence of AVN in proximal humeral fractures.

In 2015, Siebenbürger et al. published the first study that investigated the correlation between timing of surgery and the general rate of complications in operative treatment of proximal humerus fractures [16]. This study evaluated the data of 329 patients and most of them were classified as having two-part (*n* = 126) and three-part fractures (*n* = 136). In their study, 13 patients had a fracture dislocation, which was the smallest group of patients. All patients were treated with open reduction and locking plate fixation, comparable to the current study. The authors found that patients with early surgery (<48 h) and intermediate surgery (>48 h to 5 days) did not exhibit a reduced rate of complications. However, late surgery (>5 days) was associated with a higher rate of loss of fixation and AVN (odds ratio: 1.6). Overall, in 6.4% of cases, AVN of the humeral head was evident.

Two studies have been published that investigated the influence of timing on the rate of AVN after surgical reconstruction of proximal humerus fractures. Boesmueller et al. enrolled 154 patients in their study including nine patients (5.8%) with a fracture dislocation [4]. Mean time to surgery was 5.28 days (range: 0–48 days) and the authors found that time to surgery had no influence on the AVN rate. In the second study, Archer et al. evaluated the data of 19 patients without fracture dislocations [2]. The authors also did not find a correlation between time to surgery and development of AVN. It should be noted that in both studies, patients with fracture dislocations were not analyzed separately.

According to the literature, the prevalence of ischemic head necrosis after operative reconstruction of the humeral head is 3–35% [1, 7, 8, 19]. The rate of AVN in the current study is particularly high at 33%. A number of risk factors for the development of posttraumatic AVN have previously been reported in the literature. According to Hertel et al., there are three main factors that increase the risk of development of AVN in a proximal humerus fracture: fracture of the anatomical neck, integrity of the medial calcar (<8 mm), and disruption of the medial hinge [7]. In addition, Spross et al. found a correlation between fracture dislocations and increased incidence of AVN of the proximal humerus [17]. The articular surface of the humeral head has a sparse blood supply. The branch of the anterior humeral circumflex artery provides a significant proportion of the flow to the humeral head articular surface in a retrograde fashion [2]. It can be assumed that the blood supply of the humeral head is comparable to the femoral head. Here, a temporal relationship between timing of internal fixation of displaced femoral neck fractures and development of AVN has been established [12, 14]. In dislocation fractures of the proximal humerus, a shorter time to surgery may decrease the rate of AVN. The current study supports the hypothesis that there is a correlation between time to surgery and development of AVN in patients with fracture dislocation of the proximal humerus. Further studies with a larger group of patients are necessary to confirm our findings.

Secondarily, the correlation between quality of fracture reduction and AVN rate was analyzed. The quality of fracture reduction plays an important role in the treatment of proximal humerus fractures. Recently, our study group showed that anatomical fracture reduction with a locked plate significantly improved the clinical outcome of unstable and displaced proximal humeral fractures involving the anatomical neck [15]. In the current study, poor fracture reduction was associated with a 4.5-fold RR for development of AVN. This is in agreement with the findings of Bastian and Hertel, who evaluated the occurrence of AVN in proximal humerus fractures [3]. Eight of ten initially ischemic heads did not develop AVN. This was ascribed to a stable, anatomic reduction, which allowed for revascularization. In the current study, poor fracture reduction led to higher complications rates (20% vs. 65%), and patients with poor fracture reduction underwent significantly more revision surgeries (50% vs. 0%; *p* = 0.011). The number of patients with poor fracture reduction was particularly high: 20 of 30 patients. However, the proposed criteria for fracture reduction were very strictly defined. Other factors such as female gender, age >65, and more than one comorbidity did not increase the risk for complications or revision surgeries.

### Limitations

This study was limited by its retrospective design. There was no control group, and a power analysis was not performed. Owing to the small sample size, the study was purely exploratory in design, and multiple tests without adjustment for multiplicity were performed. The *p *values reported here can only be interpreted descriptively. It should be further noted that the clinical scores were investigated at final follow-up after potential revision surgery.

## Practical conclusion

- In dislocation fractures of the humeral head, early reconstructive surgery within 48 h of trauma and anatomical reduction significantly decreases the risk of AVN.
- In the case of a fracture dislocation of the proximal humerus, the operative surgeon should be aware of these facts and early surgery should be performed to decrease the risk of AVN.
